# Supplementary material for: Arterial Hypertension as a Risk Factor for Reduced Glomerular Filtration Rate after Living Kidney Donation
Source: J Clin Med. 2020 Jan 25;9(2):338. doi: 10.3390/jcm9020338 (PMC7073681; doi:10.3390/jcm9020338)
Supplement: Supplementary file 1 [file jcm-09-00338-s001.pdf]

Supplemental tables and figures.

Table S1. Univariate analysis for the development of an eGFR <60 ml/min/1.73m^2 at follow-up.

	**HR**	**95 % CI**	**p-value**
Age (years)	1.07	1.04-1.09	<0.001
Female gender	1.10	0.70-1.71	0.682
BMI (kg/m^2)	1.02	0.96-1.08	0.545
eGFR (ml/min/1.73m^2)	0.97	0.96-0.99	<0.001
Related to recipient	0.62	0.40-0.95	0.027
Smoking	1.15	0.75-1.79	0.520
Arterial hypertension	2.75	1.64-4.58	<0.001

Table S2. Univariate analysis for the development of an eGFR <60 ml/min/1.73m^2 and decrease of ≥40 % from baseline at follow-up.

	**HR**	**95 % CI**	**p-value**
Age (years)	1.10	1.05-1.14	<0.001
Female gender	1.19	0.52-2.73	0.679
BMI (kg/m^2)	1.09	1.00-1.19	0.055
eGFR (ml/min/1.73m^2)	1.00	0.98-1.03	0.893
Related to recipient	0.55	0.25-1.17	0.121
Smoking	1.54	0.71-3.33	0.276
Arterial hypertension	5.67	2.51-12.85	<0.001

Table S3. Univariate analysis for the development of an eGFR <45 ml/min/1.73m^2 at follow-up.

	**HR**	**95 % CI**	**p-value**
Age (years)	1.15	1.08-1.22	<0.001
Female gender	1.60	0.52-4.91	0.413
BMI (kg/m^2)	1.10	0.98-1.22	0.102
eGFR (ml/min/1.73m^2)	0.94	0.90-0.98	0.005
Related to recipient	0.74	0.27-2.01	0.553
Smoking	0.64	0.21-1.95	0.428
Arterial hypertension	5.08	1.76-14.71	0.003

Table S4. Univariate analysis for combined endpoint of a major cardiovascular event or death during follow-up.

	**HR**	**95 % CI**	**p-value**
Age (years)	1.08	1.00-1.16	0.019
Female gender	0.36	0.10-1.27	0.110
BMI (kg/m^2)	1.08	0.93-1.27	0.321
eGFR (ml/min/1.73m^2)	0.99	0.96-1.03	0.733
Related to recipient	2.72	0.33-22.13	0.350
Smoking	1.71	0.46-6.30	0.421
Arterial hypertension	2.99	0.62-14.55	0.175

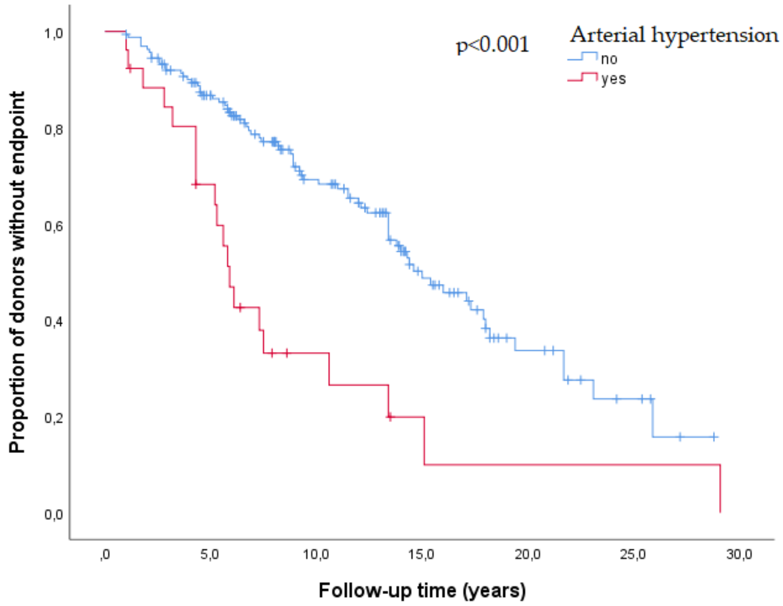

Numbers at risk	163	124	74	33	12	4
	26	15	4	1	1	1

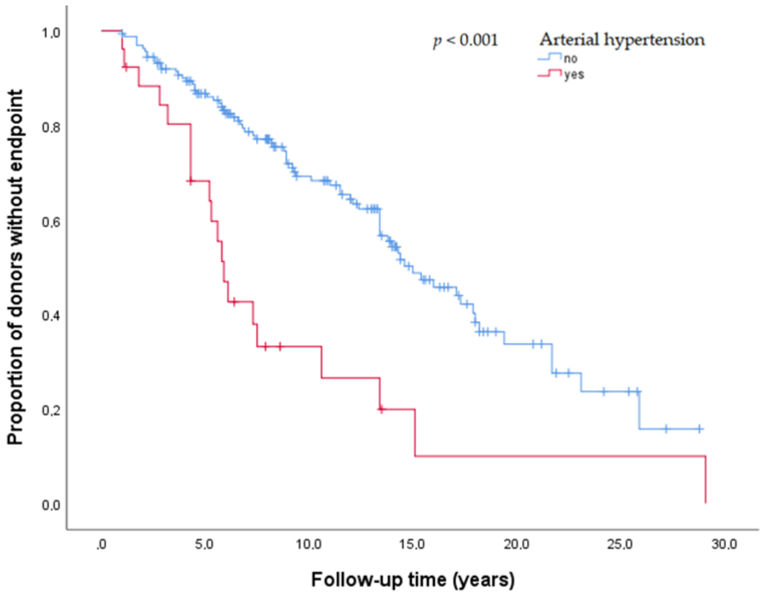

Numbers at risk	163	124	74	33	12	4
	26	15	4	1	1	1

Figure S1. Kaplan Maier plot for arterial hypertension as a risk factor for eGFR <60 ml/min/1.73m² at follow-up.

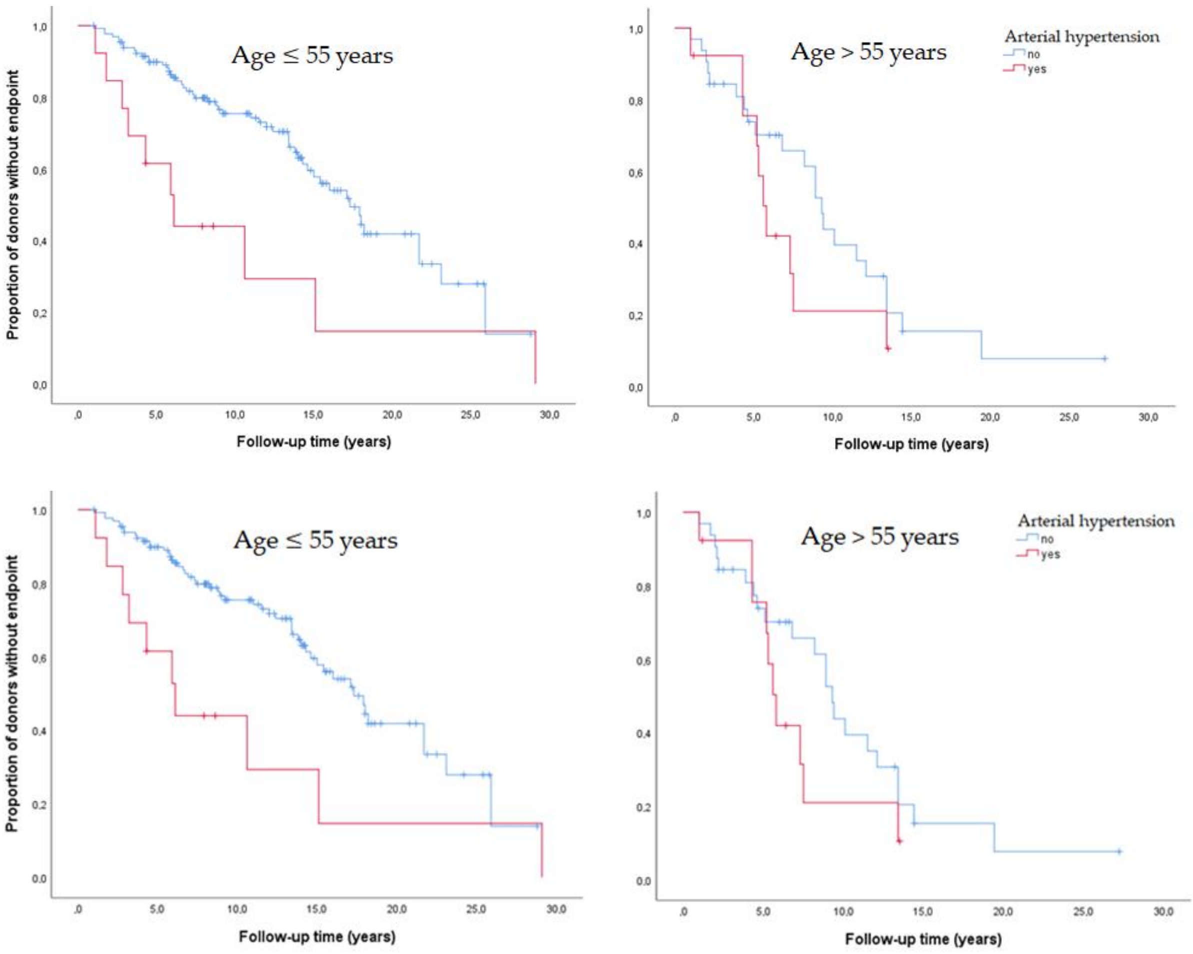

Figure S2. Kaplan Maier plot for arterial hypertension as a risk factor for eGFR <60 ml/min/1.73m^2 at follow-up, divided by age groups.
